# Supplementary material for: ENHANCED CLEAVAGE OF GENOMIC CCR5 USING CASX2Max
Source: bioRxiv. 2025 Jul 11:2025.07.08.663680. Preprint. [Version 1] doi: 10.1101/2025.07.08.663680 (PMC12265720; doi:10.1101/2025.07.08.663680)
Supplement: Supplement 1 — Supplementary Table 1 (Table S1): List of gRNA spacer sequences used. [file media-1.pdf]

Supplemental Table S1

gRNA Spacer Sequences

CasX2 sgRNA	Target Description	cDNA of Spacer Sequence	Reference
sg5 (17 nt)	nucleotide positions 272 to 288 in CCR5	AAAGTCCCACTGGGCGG	Armstrong et al. 2023
sg5 (20 nt)	nucleotide positions 269 to 288 in CCR5	AAAGTCCCACTGGGCGGCAG	Armstrong et al. 2023
sg5 (23 nt)	nucleotide positions 266 to 288 in CCR5	AAAGTCCCACTGGGCGGCAGCAT	Armstrong et al. 2023
sg10 (17 nt)	nucleotide positions 1021 to 1037 in CCR5	TGCTCCCCAGTGGATCG	Armstrong et al. 2023
sg10 (20 nt)	nucleotide positions 1018 to 1037 in CCR5	TGCTCCCCAGTGGATCGGGT	Armstrong et al. 2023
sg10 (23 nt)	nucleotide positions 1015 to 1037 in CCR5	TGCTCCCCAGTGGATCGGGTGTA	Armstrong et al. 2023
E6 (20 nt)	nucleotide positions 216 to 235 in GFP	TGTGGTCGGGGTAGCGGCTG	US Patent Application US20220220508A1
**SaCas9 sgRNA**		**Sequence**	**Reference**
CCR5-A (20 nt)	nucleotide positions 225 to 275 in CCR5	CGGCAGCATAGTGAGCCCAG	Dash et al. 2023
CCR5-B (20 nt)	nucleotide positions 1009 to1028 in CCR5	TCAGTTTACACCCGATCCAC	Dash et al. 2023
**CasX2^{Max} sgRNA in *in silico* modeling**		**Sequence**	**Reference**
CasX2 sgRNA (20 nt)	Fifth protospacer downstream of the DpbCas12e (CasX1) ORF (Accession KU516152.1)	TCCTGCAGCAGAAAATCAAA	Tsuchida et al 2022
