# Supplementary material for: ENHANCED CLEAVAGE OF GENOMIC CCR5 USING CASX2Max
Source: bioRxiv. 2025 Jul 11:2025.07.08.663680. Preprint. [Version 1] doi: 10.1101/2025.07.08.663680 (PMC12265720; doi:10.1101/2025.07.08.663680)
Supplement: Supplement 2 — Supplementary Table 2 (Table S2): List of sgRNA scaffold sequences used. [file media-2.pdf]

Supplemental Table S2

sgRNA scaffold sequences

ID	Description	Description	Reference
CasX2 sgRNA	Native CasX2 sgRNA (sgRNAv2)	GUACUGGCGCUUUUAUCUCAUUACUUUGAGAGCCAUCACCAGCGACUAUGUCGUAUGGGUAAAGCGCUUAUUUAUCGGAGAGAAAUCCGAUAAAUAAGAAGCAUCAAAG	Tsuchida et al 2022
CasX1 sgRNA	Native CasX1 sgRNA (sgRNAv1)	GGCGCGUUUAUUCCAUUACUUUGGAGCCAGUCCCAGCGACUAUGUCGUAUGGACGAAGCGCUUAUUUAUCGGAGAGAAACCGAUAAGUAAAACGCAUCAAAGUCCUGCAGCAGAAAAUCAAA	Burstein et al 2017
SaCas9 sgRNA	Native SaCas9 sgRNA	GUUUUAGUACUCUGGAAACAGAAUCUACUAAAACAAGGCAAAAUGCCGUGUUUAUCUCGUCAACUUGUUGGCGAGA	Ran et al 2015
