# Supplementary material for: ENHANCED CLEAVAGE OF GENOMIC CCR5 USING CASX2Max
Source: bioRxiv. 2025 Jul 11:2025.07.08.663680. Preprint. [Version 1] doi: 10.1101/2025.07.08.663680 (PMC12265720; doi:10.1101/2025.07.08.663680)
Supplement: Supplement 4 — Supplementary Table 4 (Table S4): List of oligonucleotides used for cloning of gRNAs. [file media-4.pdf]

Supplemental Table S4

Guide Cloning Oligonucleotides

**CasX2_sgRNA**	**forward primer**	**Reverse primer**
sg5 (17 nt), for plasmids with one guide site	CAAAGAAAGTCCCACTGGGCGGTTTTT	CGGCAAAAACCGCCCAGTGGGACTTTC
sg5 (20 nt), for plasmids with one guide site	CAAAGAAAGTCCCACTGGGCGGCAGTTTTT	CGGCAAAAACTGCCGCCCAGTGGGACTTTC
sg5 (23 nt), for plasmids with one guide site	CAAAGAAAGTCCCACTGGGCGGCAGCATTTTTT	CGGCAAAAAATGCTGCCGCCCAGTGGGACTTTC
sg5 (23 nt), for plasmids with two guide sites (site A)	CATCAAAGAAAGTCCCACTGGGCGGCAGCAT	AAAAATGCTGCCGCCCAGTGGGACTTTCTTT
sg10 (17 nt), for plasmids with one guide site	CAAAGTGCTCCCCAGTGGATCGTTTTT	CGGCAAAAACGATCCACTGGGGAGCAC
sg10 (20 nt), for plasmids with one guide site	CAAAGTGCTCCCCAGTGGATCGGGTTTTTT	CGGCAAAAAACCCGATCCACTGGGGAGCAC
sg10 (23 nt), for plasmids with one guide site	CAAAGTGCTCCCCAGTGGATCGGGTGTATTTTT	CGGCAAAAATACACCCGATCCACTGGGGAGCAC

**SaCas9_sgRNA**	**forward primer**	**Reverse primer**
CCR5-A (20 nt), for plasmids with one guide site	GAAACACCGCGGCAGCATAGTGAGCCCAGGTTT	ACTAAAACCTGGGCTCACTATGCTGCCGCGGTG
CCR5-B (20 nt), for plasmids with one guide site	GAAACACCGTCAGTTTACACCCGATCCACGTTT	ACTAAAACGTGGATCGGGTGTAAACTGACGGTG
CCR5-A (20 nt), for plasmids with two guide sites (site A)	CACCGCGGCAGCATAGTGAGCCCAG	AAACCTGGGCTCACTATGCTGCCGC
