# Supplementary material for: ENHANCED CLEAVAGE OF GENOMIC CCR5 USING CASX2Max
Source: bioRxiv. 2025 Jul 11:2025.07.08.663680. Preprint. [Version 1] doi: 10.1101/2025.07.08.663680 (PMC12265720; doi:10.1101/2025.07.08.663680)
Supplement: Supplement 5 — Supplementary Table 5 (Table S5): List of PCR primers used in Figures 1, 3, 4, 5 and 6. [file media-5.pdf]

Supplemental Table S5

Primers for PCR

Figure	Primer name	Primer Sequence	Amplicon
Figures 1B & 4A/B	CMV-pro_CAH	CAAGTCTCCACCCCATTGAC	Target
	pcDNA_CCR5_596Rev	GATAGTCATCTTGGGGCTGG	
Figures 1B & 4C/D	pcDNA_CCR5_596 For	CCAGCCCCAAGATGACTATC	Target
	CAH_bGH_Rev1	GGAAAGGACAGTGGGAGTGG	
Figures 1C, 3C & 6A	CAH_CCR5_F5	GGACAGGGAAGCTAGCAGC	Full-length genomic
	CAH_CCR5_R5	CCCCATAGCAAGACAAAGACC	
Figure 2A	ND637	TAATACGACTCACTATAGGG	Target
	ND852	GTCCACTATTAAAGAACGTGG	
Figure 3A	T7_pro_for_CAH	ACGACTCACTATAGGGAGAC	Full-length target
	pcDNA_bgh_poly_R	GACAATGCGATGCAATTTCC	
Figures 4E/G & 5	CAH_CCR5_F5	GGACAGGGAAGCTAGCAGC	Genomic
	pcDNA_CCR5_596_rev	GATAGTCATCTTGGGGCTGG	
Figures 4F/H & 5	pcDNA_CCR5_596 for	CCAGCCCCAAGATGACTATC	Genomic
	CAH_CCR5_R5	CCCCATAGCAAGACAAAGACC	
